# Sound disrupts sleep-associated brain oscillations in rodents in a meaning-dependent manner

**DOI:** 10.1038/s41598-022-09457-6

**Published:** 2022-04-11

**Authors:** Philipp van Kronenberg, Linus Milinski, Zoë Kruschke, Livia de Hoz

**Affiliations:** 1 Berlin Institute of Health and Neuroscience Research Center, Charité – Universitätsmedizin Berlin, Berlin, Germany; 2 Department of Neurogenetics, Max Planck Institute for Experimental Medicine, Göttingen, Germany; 3 Developmental, Neural and Behavioural Biology, Georg August University of Göttingen, Göttingen, Germany; 4 Present Address: Department of Physiology, Anatomy and Genetics, University of Oxford, Oxford, UK

**Keywords:** Auditory system, Circadian rhythms and sleep, Sensory processing

## Abstract

Sleep is essential but places animals at risk. Filtering acoustic information according to its relevance, a process generally known as sensory gating, is crucial during sleep to ensure a balance between rest and danger detection. The mechanisms of this sensory gating and its specificity are not understood. Here, we tested the effect that sounds of different meaning have on sleep-associated ongoing oscillations. We recorded EEG and EMG from mice during REM and NREM sleep while presenting sounds with or without behavioural relevance. We found that sound presentation per se, in the form of a neutral sound, elicited a weak or no change in the power of sleep-state-dependent EEG during REM and NREM sleep. In contrast, the presentation of a sound previously conditioned in an aversive task, elicited a clear and fast decrease in the EEG power during both sleep phases, suggesting a transition to lighter sleep without awakening. The observed changes generally weakened over training days and were not present in animals that failed to learn. Interestingly, the effect could be generalized to unfamiliar neutral sounds if presented following conditioned training, an effect that depended on sleep phase and sound type. The data demonstrate that sounds are differentially gated during sleep depending on their meaning and that this process is reflected in disruption of sleep-associated brain oscillations without behavioural arousal.

## Introduction

Sleep places animals at risk due to the accompanying decreased behavioural response to sensory stimuli^1,2^ and the associated species-specific sleep posture^3^. This risk is a trade-off to carry essential physiological processes^4–7^ such as brain homeostasis/restoration processes^8–10^ or memory consolidation^11^. Sensory disconnection during sleep is not complete, however, and some continuation of sensory processing remains^12^ in order to detect behaviourally relevant stimuli. The mechanisms and specificity of this continued sensory processing are not understood. Here we explore the role of meaning in the effect that sound has on sleep-associated brain oscillations in the rodent brain.

Sensory processing of stimuli is reduced in the sleeping brain across modalities (olfactory^13,14^, visual^15^ and somatosensory^16,17^) with the exception of the processing of sounds by different auditory stations, including the midbrain^18^ and primary cortex^19^. That sounds are treated differently from other stimuli may not be surprising since sounds, fast carriers of information about sources that are far and around^20^, are valuable in the detection of approaching dangers. Here, in order to better understand the mechanisms and selectivity of sound processing during sleep, we tested the effect that neutral and danger-predicting sounds had on mouse sleep-associated ongoing brain oscillations during different sleep phases. Other animals, especially cats have been used to investigate arousal thresholds^21^ and habituation^22,23^ during sleep, but we have little understanding about the role of sound meaning in these processes^24^.

We developed a paradigm, where an auditory associative learning task was combined with chronic electrophysiological recording during subsequent sleep. During conditioning, a sound was associated with an aversive experience. This sound, as well control sounds of neutral meaning to the animal, were then presented to the mouse during sleep. Ongoing brain oscillations, measured through EEG, were the readout to determine if a sound had an effect on the sleeping brain. The combined behavioural/EEG paradigm is useful for the investigation of the circuit regulating sensory gating in the sleeping and, potentially, waking brain. Sensory gating, the filtering of stimuli according to their relevance^25^, is crucial in our everyday life, and especially important to achieve a continuous sleep^26^. Hence, sleep is an effective model to study sensory gating mechanisms.

## Results

**Figure 1:**
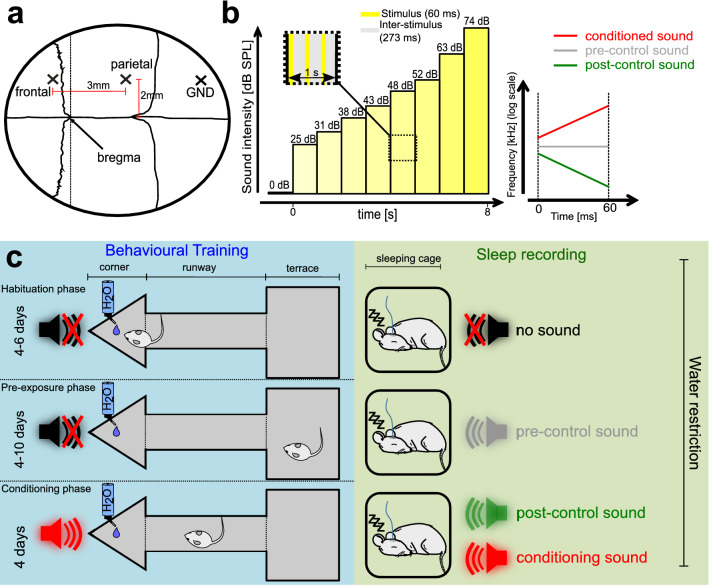
Experimental design. (**a**) Sketch of mouse skull with implantation sites for parietal/frontal EEG and ground screw. (**b**) Left: Stimulus architecture over time. Insert shows stimulus and inter-stimulus time intervals within each intensity block. Eight intensity blocks (zero intensity excluded) each with three pulses adding up to 24 pulses over eight seconds. Right: Schematic of frequency modulated conditioned and post-control sound and pure tone pre-control sound. (**c**) Experimental Paradigm. Left (blue): Behavioural training on the audio terrace, consisting of corner, runway and terrace. Unlimited water access and no sounds in the corner during habituation and pre-exposure phase. During the conditioning phase, water access is granted for 20–40 s, before a conditioning trial starts. Right (green): Sound exposure during NREM and REM phases over the experimental stages.

To study sound processing during sleep, we first played meaningful and neutral sounds during different sleep phases to nine female C57BL/6J mice. First, during a 6-day long ‘exposure phase’, we recorded the EEG and EMG activity in sleeping mice to identify REM (rapid eye movement) and NREM (non REM) phases of sleep for 1–2.5 h per day. During sleep, we played a fixed ‘pre-control’ pure tone for 8 s with increasing intensity to assess the effect that a neutral sound has on different sleep phases. During the following 4–8 days, we trained animals to escape an aversive stimulus (loud broadband noise plus air puff) that was predicted by a conditioned frequency-modulated (FM) sound. As before, during subsequent 1–2.5 h-long sleep sessions, we identified REM and NREM phases of sleep, and played the conditioned and an unfamiliar neutral FM sound (post-control sound), in increasing intensity (Fig. 1b), for 8-s during either sleep phase. The post-control sound was introduced, as a neutral but also novel sound, to dissociate effects of the conditioned sound that resulted from its valence as a predictor of punishment from those that resulted from its novelty during sleep.

### Manual sleep phase classification matched offline spectral analysis

**Figure 2:**
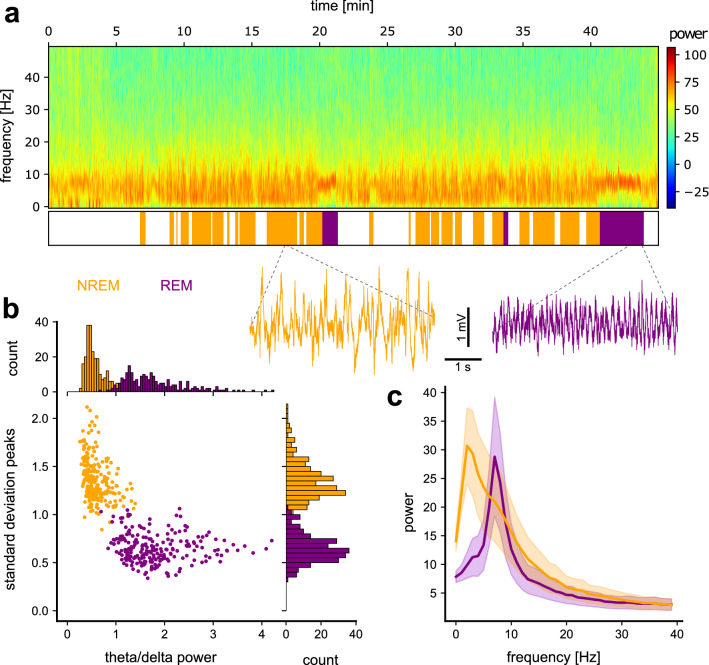
Sleep stage characterization. (**a**) Top: Power spectrogram of an example parietal EEG recording including sleep. Middle: Visual sleep scoring (based on EEG + EMG; awake = white, NREM = orange, REM = purple) of the spectrogram shown above. Bottom: two example EEG traces taken from a typical NREM (orange)/REM (purple) sleep bout. (**b**) The scatter plot depicts the theta/delta frequency bands power ratio against the peak-to-peak variability (standard deviation). Data from undisturbed sleep. The histograms indicate the distributions of each measure for NREM (orange) and REM (purple) sleep. (N (animals) = 11, n (sleep sections) = 246 (NREM), 228 (REM)) **(c)** Mean spectral power distribution across frequency for NREM and REM sleeps. (N (animals) = 11, n (sleep sections) = 246 (NREM), 228 (REM)).

During the experiment, we used online visual inspection, based on conventional criteria in sleep scoring^27,28^, of the ongoing oscillations to categorize the current vigilance state as awake, REM, NREM, or unidentifiable. To ensure that this decision matched a quantifiable measure, we first compared the manual scoring with a quantitative analysis of the corresponding EEG spectra. For this analysis, we used eight second EEG snippets (undisturbed sleep without sound presentation) recorded from eleven animals (9 from FM experiment described above + 2 generalization experiment described below) during the first 4 days of pre-exposure and conditioning phases. Overall, this data set comprised a total of 474 recorded sleep snippets with 246 scored as NREM and 228, as REM. We found that the experimenter’s scoring paralleled the spectrogram of the parietal EEG (Fig. 2a). Phases that had been categorized as NREM sleep were characterized by synchronized delta activity (delta frequency band, 0–4 Hz) and high amplitude irregular activity typical of NREM (Fig. 2a, orange trace). In contrast, phases categorized as REM sleep were characterized by fast and desynchronized activity (theta band, 6–10 Hz) with lower amplitude and high regularity (Fig. 2a, purple trace) as described by Jouvet and collegues^29^. A quantification of both the theta/delta power ratios and the peak-to-peak variability in the amplitude in 4-s long time windows, clearly separated manually classified NREM (orange) and REM (purple) phases into segregated clusters (Fig. 2b). For sections scored as REM sleep, we found strong theta activity (Fig. 2c) and regular peak-to-peak amplitude. Compared to REM, NREM sections had low theta/delta power ratios (Fig. 2b) and high peak-to-peak standard deviation, as expected from typical NREM activity. To conclude, the quantification of the EEG signal validated the subjective characterization of NREM and REM sleep performed during the experiment.

### The conditioned sound elicits reductions in state-dependent EEG power during NREM and REM sleep

**Figure 3:**
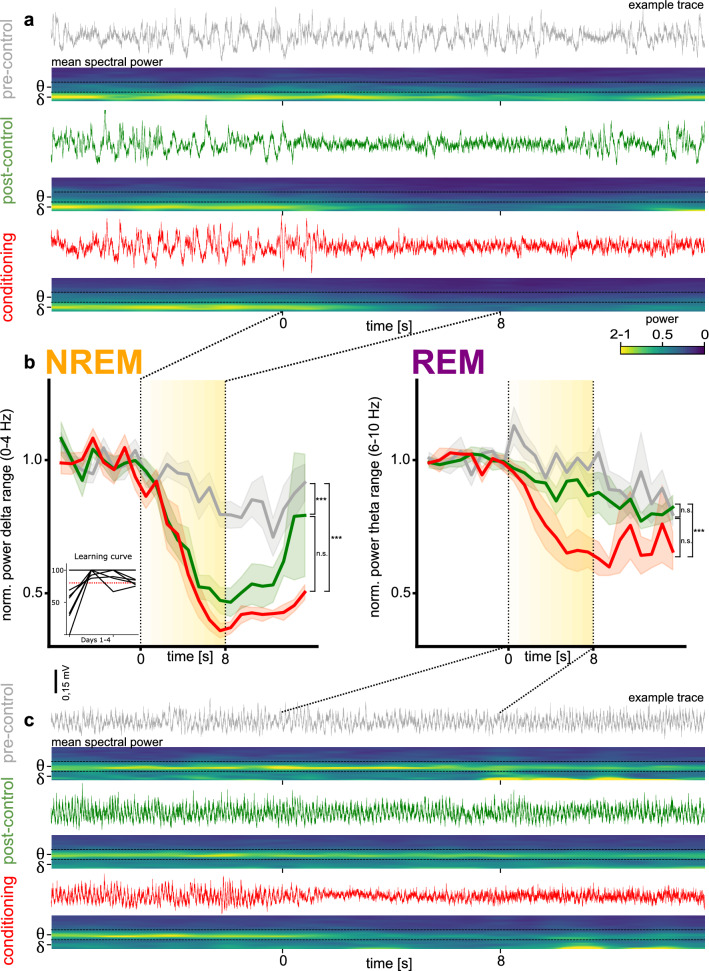
Sound-driven EEG power changes during NREM and REM sleep. (**a**) NREM. Top traces: Example EEG traces. Bottom spectrograms: mean power spectrograms during NREM sleep over animals and sleep sections before, during and after each sound type played. δ = Delta frequency band (0–4 Hz), θ = Theta frequency band (6–10 Hz). Time points 0 and 8 mark the beginning and end of sound presentation respectively. (**b**) The mean normalized EEG power in the delta (for NREM) and theta (for REM) ranges over time for learner animals (> 80% correct trials on second day and >= 15 trials in total) as the pre-control (grey), post-control (green), and conditioned (red) sounds were presented. Sound presentation is illustrated by yellow block with sound intensity reflected in the color intensity. Significance level is indicated as follows: *p < 0.05, **p < 0.01, ***p < 0.001, (N = 6 (trials = 246 (NREM), 228 (REM)). (**c**) REM. Same as (**a**), but for REM sleep.

To quantify changes in the state-dependent EEG elicited by sound presentation, we analysed the dynamics of power in the delta frequency band (0–4 Hz) for NREM sleep and in the theta frequency band (6–10 Hz) for REM sleep in the 9 mice conditioned to FM sounds. The power was calculated on a second-to-second basis for each individual EEG trace over 24 s (eight seconds before sound onset, eight seconds during sound presentation at increasing intensity, and eight seconds after sound offset). We used EEGs recorded during the first 4 days of the exposure phase and first 4 days of the conditioning phase. We separated the mice in 6 learners, with more than 80% avoidance on day 2 of conditioning, and 3 non-learners (less than 80% avoidance or less than 15 conditioning trials). Visual inspection revealed that there were differences in the effect of pre-control, conditioned and post-control sounds on both NREM and REM sleep (Fig. 3a–c) in learners. Further frequency ranges and the effect the sound has on them can be found in Fig. S1.

Generally, power changes were weaker during REM sleep (theta power) than during NREM sleep (delta power). Upon presentation of the conditioned sound during NREM sleep, power dropped on average to ~ 35% of baseline values before sound onset, whereas during REM sleep power dropped to ~ 60%. The same was true for the other experimental sounds, which elicited power changes that were different in size between NREM and REM. We used linear mixed models separately for the 8 s before (block 1), during (block 2) and after (block3) sound presentation, to separate the baseline activity, power changes during sound presentation and power dynamics after the sound ceased. We did not find any effect for block 1, as expected, because it represents the baseline activity before the experimental sounds were presented. A linear mixed model during block 2 with sleep phase, sound, time and interaction between sound and time as fixed effects, and animals and session as random effects, revealed a main effect of phase (F(1, 2105) = 26, p < 0.001, EES (Estimated effect size) = -0.117), time (F(1,2105) = 4.94, p < 0.05) and sound-time interaction (F(2,2105) = 18.8, p < 0.001). Time was defined from 1–8 s for each block and sessions represent each individual sound presentation within sleep phases and animals. Including session and animals as random effects significantly improved the fit of the model using a likelihood ratio test (X^2^ (6) = 639.2, p < 0.001). The sound-time interaction disappeared in block 3. We found phase (F(1,2105) = 50.38, p < 0.001) and sound (F(2,2105) = 13.96, p < 0.001) to be the relevant fixed effects during block 3, with the estimated effect for phase (− 0.223) doubling in strength with respect to block 2.

In a raw EEG example trace recorded during NREM sleep, one can observe that the typical delta activity, which had not been influenced by the pre-control sound (Fig. 3a top), was disrupted by both the post-control and conditioned sounds (Fig. 3a mid and bottom). This difference in effects is also evident in the mean spectral power across mice over the first 4 days of conditioning, with superimposed mean for the first 4-days of exposure (Fig. 3b left, red and grey). The power in the delta range (0–4 Hz) did not change with pre-control sound presentation but decreased drastically as the conditioned and post-control sounds were played (Fig. 3b left). This power decrease was temporary and lasted typically for 4–8 s after sound offset. This data set comprises of the following number of sleep bouts: [sound/sleep type *range* across animals (mean)] pre-control/NREM *2–15* (8.33), post-control/NREM *5–11* (7.84), conditioned/NREM *4–10* (7.5). Only animals that learned the task were included here (6/9 mice; over 80% avoidance on the second day and at least 15 conditioning trials by end of day 2, Fig. 3b, inset. The slight drop in power towards the end of the pre-control sound presentation coincided with the loudest intensities of the sound sequence. In contrast, for the post-control and the conditioned sound, the drop in power was stronger and already very prominent during the lower intensity repetitions of the sound sequence, at intensities between 30 and 40 dB SPL. That the post-control but not the pre-control sound elicited a change comparable to that of the conditioned sound suggests some form of generalization around the conditioned sound in the sensitivity to sound during NREM. A linear mixed model of block 2 during NREM sleep with sound type, time, and their interaction as fixed effects and animal and session as random effects, revealed a main effect of time (F(1, 1130) = 4.49, p < 0.05) and sound-time interaction (F(2, 1130) = 20.51, p < 0.001). More specifically both, post-control (EES = − 0.049, p > 0.001) and conditioned sounds (EES = − 0.07, p > 0.001) had significant interactions with time, as expected given the strong power change both elicit during block 2. During block 3 we found a main effect sound (F(2,1130) = 8.81, p < 0.001) driven by the post-control (EES = − 0.367, p < 0.001) and conditioned (EES = − 0.392, p < 0.001) sounds. But unlike the conditioned sound, the effects of the post-control sound showed a tendency to diminish over time (p = 0.064).

During REM, the pattern was somewhat different. As can be seen in the example traces (Fig. 3c), only the conditioned sound elicited a change in the EEG pattern. Quantification of the mean 6–10 Hz theta power across mice confirms this pattern (Fig. 3b right). Only the conditioned sound elicited a strong power change during REM. Generalization of the effect to the post-control sound was either absent or weak during REM sleep compared to NREM sleep. This data set was comprised of the following number of sleep bouts: [sound/sleep type *range across animals* (mean)]: pre-control/REM *2–8 (*4.67), post-control/REM *3–11* (8.17), conditioned/REM *3–11* (7.5). A linear mixed model of block 2 with sound type, time, and their interaction as fixed effects and animals and sessions as random effects, revealed a main effect of sound-time interaction (F(2, 970) = 8.09, p < 0.001), which was driven only by the conditioned sound (EES: − 0.036, p < 0.01) as is evident from the plot (Fig. 3B, right). During block 3 we found a main effect of sound (F(2,970) = 7.95, p < 0.001) with no interaction with time, which is driven again only by the conditioned sound (EES:-0.356, p < 0.001).

Sound presentation was occasionally accompanied by muscle twitches, which were visible in the EMG. No full awakening took place, however, as reflected in the EEG pattern after sound-driven disruption. Nevertheless, the change in EEG pattern to a more desynchronized pattern during NREM sleep and to a more irregular pattern during REM sleep, point in the direction of a more unstable/alert state of the brain.

Overall, the pre-control sound led to subtle reductions in power that were small and late relative to sound onset. In contrast, the conditioned sound elicited strong and fast reductions in state-dependent EEG power during both NREM and REM sleep. Interestingly, this effect was generalized to the post-control sound, albeit with a weaker effect, only during NREM sleep.

### Power change effects are strongest during early conditioning days

**Figure 4:**
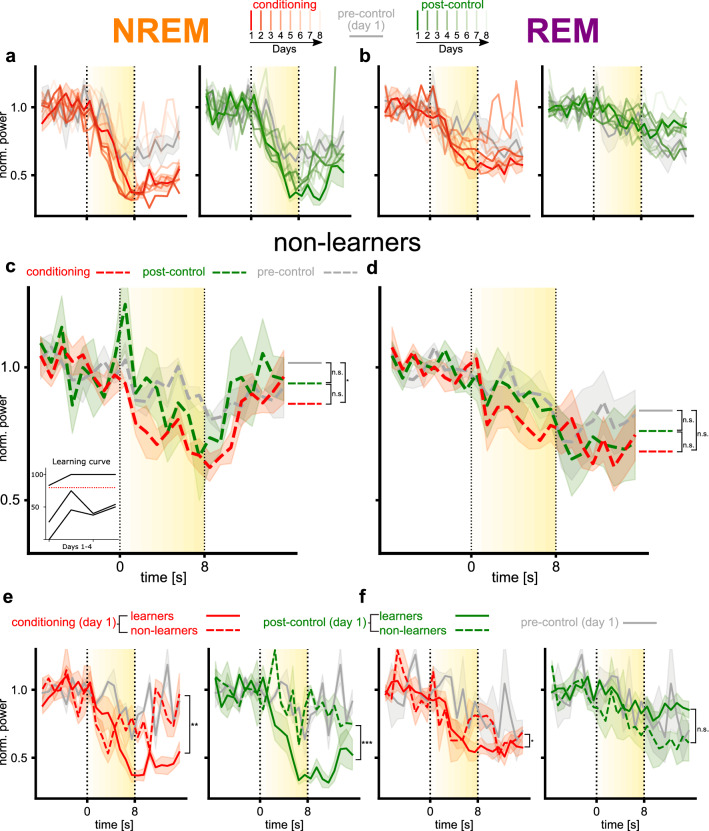
Effect of day and learning rate on EEG power changes. (**a**,**b**) Comparison of mean daily EEG power changes across animals during (**a**) NREM sleep and (**b**) REM sleep for the conditioned (red) and post-control (green) sounds. Changes are measured over eight consecutive days with decreasing color strength, starting on conditioning day 1. Responses to pre-control sound on the first day serve as reference. (N = 6). (**c**,**d**) Mean EEG power in (**c**) delta range for NREM and (**d**) theta range for REM over time for non-learner animals (< 80% correct trials on second day or < 15 trials in total) as the pre-control (grey), post-control (green), and conditioned (red) sounds were presented. Sound presentation is illustrated by a yellow block with sound intensity reflected in the color intensity. Significance level is indicated as follows: *p < 0.05, **p < 0.01, ***p < 0.001. (N = 3). (**e**,**f**) Comparison of EEG power changes during (**e**) NREM sleep and (**f**) REM sleep phases of the first recording session after conditioning. Learners (≥ 80% correct trials, solid line) and non-learners (< 80% correct trials, dashed line) are defined by second day behavioural performance in the conditioning paradigm. Responses to pre-control sound on day 1 of exposure (averaged over animals) serve as reference.

To assess whether the observed effects changed as the sounds became more familiar over the course of days, we analysed the stimulus effects on sleep effects on a daily basis over the first 8 days of conditioning (Fig. 4a,b). Sound presentations differed between animals over days, because of individual sleep differences. Each day is represented by the mean of a minimum of 3/6 mice (mean of 4.3, not all mice were trained more than 4 days) and a minimum of 4 recordings (mean of 9).

We found a decreased effect for the conditioned sound during NREM sleep over days. A linear mixed model for block 2 with day and time as interacting fixed effects and session and animals as random effects during REM sleep while the conditioned sound was played, revealed a main effect of time (F(1, 600) = 36.3, p < 0.001). During block 3 we found a main effect of day (F(7,600) = 5.06, p < 0.001), which was driven only by days 7 (EES: 0.325, p < 0.001) and 8 (EES: 0.224, p < 0.01), suggesting that the effect was stable over the first 6 days. The pattern was similar for the post-control sound with a main effect of time during block 2 (F(1, 608) = 24.12, p < 0.001), but no effect of day during block 3.

During REM sleep, we found differences between the post-control and conditioning sound regarding the effect of day. While we found that days 7 (EES: 0.057, p < 0.01) and 8 (EES: 0.05, p < 0.05) showed a significant interaction with time for the conditioned sound during block 2, we did not find any interactions between day and time for the post-control sound. For the post-control sound we surprisingly found an overall effect of day (F(7, 600) = 2.08, p > 0.05) already during block 2. The effect of day was driven by days 3 (EES: − 0.15, p < 0.05) and 4 (EES: − 216, p < 0.01), suggesting a more irregular effect of day during REM sleep compared to NREM sleep. In contrast to our findings for NREM sleep, we did not find any effects of day for either conditioned or post-control sound in block 3.

### Non-learners show no distinct EEG changes to any experimental sound

Three out of the 9 mice trained achieved, on day 2, less than 80% avoidance or completed less than 15 conditioning trials. To study how these non-learners (Fig. 4c, inset) differ from learners over the first four days of conditioning, we analysed their EEG as we did for the learners in Fig. 3b. The number of recordings included in the analysis is as follows [sound/sleep type *range across mice* (mean)]: pre-control/NREM *7–11* (8), post-control/NREM *2–8* (5), conditioned/NREM *4–8* (5.67), pre-control/REM *7–8 (*7.33), post-control/REM *4–6* (5.33), conditioned/REM *3–10* (6.33).

Compared to learners, sound in non-learners elicited no clear effect in overall EEG power (Fig. 4c,d). No significant effects were found during block 2 for NREM and REM. Nevertheless, EEG pattern changes during NREM and REM sleep for non-learners were significantly different from each other during block 3, right after the sound presentation. A linear mixed model during block 3 with phase, sound, time and interaction between sound and time, and animals and session as random effects revealed a main effect of phase (F(1, 897) = 7.67, p < 0.01, EES (Estimated effect size) = 0.109). Additionally, when looking at sound-dependent power decreases during NREM sleep in block 2 and 3 there was an effect of the post-control and conditioned sound respectively.

Hence, the responses of the non-learners were less specific and delayed compared to learners. The differences between power changes of learners and non-learners emphasize the finding that the acquired meaning of a sound determines its effect on sleep.

Interestingly, the behavioural performance on day two, which determined whether the animal was classified as ‘learner’ or ‘non-learner’, could be predicted based on the strength of the sound effects on day 1 during NREM (Fig. 4e) and for the conditioned sound during REM (Fig. 4f). We fitted a linear mixed model with learning and time as interacting fixed effects and sessions and animals as random effects. Learning is a binary variable based on the learning criteria described above. We found learning to be a reliable predictor of the effect during the first night, during NREM sleep we found for both conditions different decreasing slopes for block 2 (conditioned: F(1,132) = 4, p < 0.05, post-control: F(1,108) = 6.68, p < 0.05) and a difference between learning and non-learning animals during block 3 (conditioned: F(1,132) = 7.65, p < 0.01, post-control: F(1,108) = 36.58, p < 0.001). During REM sleep, we found a less clear picture with learning being a significant factor during block 2 for the post-control sound (F(1,164) = 5.89, p < 0.05) and during block 3 for the conditioned sound (F(1,180) = 4.91, p < 0.05), however in different directions.

In summary, learners showed pronounced EEG pattern changes during conditioned and post-control sound presentation during NREM, whereas in non-learners, weaker or slower changes were observed. Interestingly, the times scale of the effect of the post-control sound in learning animals was different to the conditioned sound (Fig. 3b), since the power change started comparatively late, only with the presentation of the loudest repetitions of the sound sequence.

### Sound generalization during NREM and REM sleep

**Figure 5:**
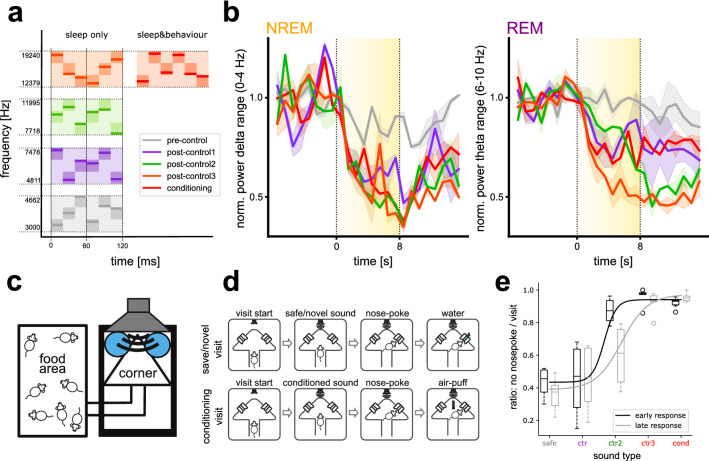
Sound generalization—EEG power changes to sound clouds. (**a**) Sound cloud architecture for all experimental sounds. Sound clouds were created pseudo randomly from three frequency subranges within the wider sound-specific frequency range. The conditioning and post-control 3 sound had the same frequency range, but differed in tone sequence. (**b**) Mean EEG power in delta (for NREM, left) and theta (for REM, right) ranges across mice over time for learner animals (> 80% correct trials on second day and > 15 trials in total). The five colors represent the five experimental sounds as in (**a**). Sound presentation is illustrated by a yellow block with sound intensity reflected in the color intensity. (N = 2). (**c**) Audio box sketch with food area, tunnel, and water-corner where the experimental sounds are presented. Figure taken from Chi et al.^30^. (**d**) Schematic of safe/novel and conditioned visits to corner. Figure taken from Chi et al.^30^^.^ (**e**) Behavioural performance in Audiobox. Percentage avoidance of nose poking/corner visit depending on sound played. The black line (first two days of exposure, early response) and the grey line (last two days of exposure late response) represent sigmoidal fits of the data. (N = 6).

To explore further the generalization between conditioned and post-control FM sounds that we observed during NREM (Fig. 3b), we tested additional sounds with a spectrotemporally richer sound architecture. We trained two female C57BL/6J mice on the audio-terrace as before. Unlike in the first experiment, we used three instead of one post-control sound. Additionally, in contrast to the sounds used in the first experiment, we used sound clouds: a concatenation of pure tones pseudo randomly selected between set frequency borders (pre-control: 3000–4662 Hz, post-control 1: 4811–7478 Hz, post-control 2: 7718–11,995 Hz, post-control 3 and conditioned: 12,379–19,240, Fig. 5a). This allowed us to rank sounds according to their similarity in frequency range to the conditioned sound. The most similar post-control sound consisted of pure tones in the same frequency band as the conditioned sound, but was discriminable by the tones’ order. The sound cloud furthest away in frequency from the conditioned sound was used as the pre-control sound.

As before, different sounds elicited different changes during NREM and REM sleep (Fig. 5b). In a linear mixed model featuring sound type and time as interacting fixed effects and variation over sessions and animals as random effects, we found an effect of sound for all sounds during block 3 of NREM (F(4,374) = 8.65, p < 0.001) and REM (F(4,382) = 9.12, p < 0.001) sleep. Additionally, we found an interesting interaction between sound and time during block 2 of REM sleep (F(4,382) = 5.84, p < 0.001), which was driven by post-control 2 (EES:-0.04, py0.01) and post-control 3 (EES:-0.058, p < 0.001) sound, the closest to the conditioned frequency. These two sounds had the clearest decrease of power during block 2, with the post-control 3 showing the earliest and strongest power drop. The pre-control sound had weak or no effect on ongoing oscillations and was significantly different from all other experimental sounds during both NREM and REM sleep (Fig. 5b). Consistent with our previous results, we did not find a clear difference between the effects of the conditioned and post-control sound clouds during NREM sleep, and all sounds elicited strong effects that did not differ from each other (Fig. 5b). This time, however, we saw generalization also during REM sleep such that the post-control 3 sound cloud, in the same frequency band as the conditioned sound cloud, elicited the strongest changes, which were significantly different from the effects of all other experimental sounds. Effects were generally weaker during REM sleep than NREM sleep, however, consistent with the previous experiment.

To assess whether the strong generalization across sound clouds during sleep was due to an inability of the mice to discriminate between these sounds, we conducted a sound discrimination experiment in a different group of mice in the Audiobox, an automatic behavioural paradigm (Fig. 5c; explained in detail in de Hoz and colleagues^31^), using the same sounds. Mice were trained to access water in the water-corner through nose-poke holes (Fig. 5d). Whenever an animal entered the water-corner, a safe sound (the pre-control sound cloud) was played for the duration of the visit to the corner, and the mouse was allowed to nose poke and thereby access water (Fig. 5d, safe visit). After this habituation phase, the conditioned sound cloud was introduced in 5% (then 10%, then 20%) of water-corner visits. If the animal nose-poked during these visits, it would receive an air-puff and no access to water (Fig. 5d, conditioned visit). Mice typically avoided nose poking in about a third of the safe visits, but quickly learned to avoid nose poking in most conditioned visits. When conditioned responses were stable (> 80% avoidance of nose pokes in conditioning visits and > 50% nose poking in safe visits) we sequentially introduced the other three novel sound clouds in 20% of visits each over 5–7 days.

The ratio of nose pokes per visit for each sound type was an indication of whether a novel sound was perceived more like the safe or the conditioned sound. To account for possible learning effects, we separated the avoidance response into early and late response according to the day of training, first two days and last 2 days respectively. Interestingly, the discrimination shifted subtly between early and late response. An ANOVA revealed a main effect of sound for the early (F(1,25) = 30.99, p < 0.001) and late (F(1,25) = 23.44, p < 0.001) responses. The post-control 1 sound, the closest in frequency to the safe sound, was consistently treated as safe by the animals (Fig. 5e). Post-hoc multiple comparisons (Tukey–Kramer) revealed that the post-control 2 sound elicited avoidance during the early response, like the unsafe sound (p = 0.9517), but led to more nose pokes later (differently from the conditioned sound, p < 0.01). Overall, the data demonstrate that the sound clouds used are behaviourally discriminable and that the broad generalization across clouds observed in the sleep experiment is not the result of a lack of discriminability. Differences in sensory gating and generalization between sleep and wakening are not well understood and require further investigation.

## Discussion

We set out to investigate whether the meaning of a sound determines its influence on sleep-associated ongoing oscillations. We found that a frequency-modulated sound previously conditioned to an aversive outcome disrupted the EEG pattern during both NREM and REM sleep, leading to a decrease in state-dependent EEG power. These changes were observed at relatively low sound intensities of 30–40 dB SPL, not loud enough to elicit awakening or startle. That these changes are related to the aversive meaning of the sound is derived from the observation that the conditioned FM sound elicited stronger changes than a neutral sound presented in the same sleep bout during both NREM and REM sleep. The effect of the neutral sound during NREM sleep was somewhat weaker than that elicited by the conditioned sound and had a tendency to be more transient. It was likely the result of generalization to the conditioning situation, since a neutral sound presented before conditioning training began had no effect on either NREM or REM EEG power. We investigated the generalisation effect further using multiple neutral spectrotemporally rich sound clouds with varying frequency distance from the conditioned cloud. We found that generalization could indeed be broad, such that sound clouds of frequencies that do not overlap with the conditioned sound also elicited changes in NREM EEG patterns. This was not related to the discriminability of the sound clouds, since mice trained to discriminate between these sounds could do so.

Thus, meaningful and neutral sounds affect distinct sleep stages differently. The effect that a sound has on sleep-associated ongoing oscillations does not depend only on its meaning but also on the sleep stage and day of conditioning. Acquired behavioural relevance increases the potential of sounds to elicit changes during sleep in mice, which is in line with the findings in humans^32^, cats^33^and rats^34^. What do these EEG pattern changes mean? The EEG can give insight into global brain processes that are, nonetheless, dissociable from behavioural responses. For example, while we found clear changes to the EEG pattern upon sound presentation, we did not observe clear behavioural responses in the form of arousal. Early studies concluded that behaviourally significant sounds are more likely to arouse an animal than a neutral sound^33,34^. Yet none of these studies has provided a quantitative evaluation of EEG changes during sleep. Our study, by measuring changes in the EEG pattern using sound intensities that do not yet elicit measurable global arousal, provide a novel and more detailed perspective on auditory sensory gating during sleep. Nevertheless, the observed EEG pattern changes (decreased EEG delta power during NREM sleep and decreased theta band power during REM sleep) indicate a transition into lighter sleep or a more alert state. It is possible that the observed depression in EEG power resembles localised arousal that prepares the brain for transition into the waking state, while at the same time not yet disrupting sleep. Delta activity (0–4 Hz EEG activity) has been suggested to be involved in sensory disconnection during sleep^35^, partially because associated off-states in neuronal firing may interfere with cortical signal propagation^36^. Thus, local or global depression in delta activity could indicate a lowering of the waking threshold. In order to fully exclude a transition to a state of quiet wakefulness, a more comprehensive characterization of physiological parameters such as respiration, heart rate, pupil size or detailed motor movement would be necessary. In future studies, these would offer useful measures to assess the role of the observed EEG pattern changes in global arousal. Moreover, it might be interesting to further characterize NREM sleep in mice to identify similar sub-stages as are well known for humans^37^ or beginning to be recognised in rats^38^. For this purpose it would be interesting to analyse longer time periods before and after the sound presentation and to quantify sleep spindles and slow oscillations. That the effect of the conditioned sound on NREM EEG patterns is strong and reliable suggests, however, that the effect is not sensitive to subtle differences within NREM.

The post-control sound was a frequency modulated sound like the conditioned sound but of opposite direction of modulation, and diverging frequency range. In addition, unlike the conditioned sound, it was never heard during wakefulness. Despite these differences, it succeeded in eliciting changes in ongoing oscillations during NREM, albeit weaker and with a tendency to be less lasting than those elicited by the conditioned sound. Future studies need to determine the parameters and sound architectures that lead not only to the sound effects in power but also to this generalization during NREM. Similarly, we will need to ascertain whether a different post-control sound, for example, one that was also presented during the wakeful experience in a neutral manner, might had favoured generalization during REM. Similarly, the possible contribution of the loud unconditioned sound to the generalization during sleep will have to be investigated.

Another main finding was that the EEG pattern changed generally more during NREM compared to REM sleep. This is consistent with higher sensory thresholds during REM sleep^33,34^. We used different oscillation frequency ranges to measure the oscillatory power for NREM and REM, which makes the changes in power across both states not fully comparable. Yet, the neutral sound presented during the sleep recording had barely any effect on REM sleep whereas, in NREM sleep, its effect was similar to that of the conditioned sound, indicating that these two phases differ qualitatively in how they gate sensory information.

Besides the different potential to depress state dependent EEG power between conditioned and control sound we also found differences in the extinction of this effect over days. Even though the animals were conditioned every day before the recording session and the conditioned sound must have been highly familiar to the mouse, the decrease in power elicited by the conditioned sound remained profound and stable over the first 6 days during NREM sleep. In contrast, the effect of the control sound shows high variability during NREM sleep already during the first days of conditioning. This suggests that the generalization over frequency-modulated sounds was not influenced by the actual strength of the response to the conditioned sound itself. Interestingly, the effect elicited by the conditioned sound on REM sleep weakened over days, again supporting our conclusion that the effect on different phases is qualitatively different.

Another interesting aspect addressed in this study is the connection between wake behavioural performance and the potential of sounds to depress state-dependent EEG power. In animals that did not learn the task, sounds did not elicit EEG power changes as compared to ‘learning’ animals. In fact, the pronounced difference between learners and non-learners observed after the first conditioning session (before the behaviour could be classified as ‘non-learner’) predicted behavioural performance on the next day. While our study was not designed to investigate targeted memory reactivation^39,40^, sound presentation during sleep might have contributed to enhance learning rate as a by-product. It would be interesting to compare our current learning rates with those of mice, which had not been exposed to the experimental sounds during sleep. Although, it is possible that our task, as designed, is learnt too quickly to facilitate the observation of learning rate differences.

Since we found that the effect of the conditioned sound generalized to the neutral sound during NREM sleep, we assessed the scope of this generalization using spectrotemporally richer sounds in the form of sound clouds. We replicated the earlier finding of the control sound presented before the conditioning phase (pre-control sound) not eliciting EEG pattern changes. For the neutral sounds presented during the conditioning phase, the generalization was broad in that all sound clouds elicited some EEG power change during both NREM and REM, independently of their frequency. Interestingly, the neutral sound that was in the same frequency range as the conditioned sound, albeit with a different temporal architecture, elicited even stronger changes during REM sleep than the conditioned sound itself. The broader generalization was not caused by a lack of discriminability of the sounds, since another group of mice could distinguish between them in a sound discrimination task. These findings suggest first, that the generalization of the effect that sounds have on REM sleep might depend on the nature of the sounds themselves, as well as their familiarity. Second, these findings suggest that the discriminability of sounds in the awake behaving animal does not automatically predict discriminability in the sleeping brain. It may be that sound features other than frequency, influence sensory gating in the sleeping brain^41^. The criteria the sleeping brain applies in stimulus interpretation and the potential to shape generalisation during sleep remain to be addressed. Finally, it will be interesting to determine the auditory circuit(s) responsible for the difference in generalization during NREM and REM sleep. Since the meaning of the sound was learned, the auditory cortex is a prominent candidate. We believe that also the feedback connection to subcortical auditory structures might be of interest^42^ For example, the inferior colliculus has been implicated in promoting wakefulness ^43^.

In conclusion, the meaning of a sound is a strong determinant of the effect this sound has on sleep-associated ongoing brain oscillations, even before any behavioural effect in the form of arousal can be detected. This effect is stronger, more persistent, and more generalizable to other sounds during NREM sleep. The paradigm presented here can be used as a model system to further explore sensory gating mechanisms and the underlying circuits involved.

## Materials and methods

### Animals

All experiments were aligned with the ethical regulations provided by the “Niedersächsisches Landesamt für Verbraucherschutz und Lebensmittelsicherheit (LAVES)”. Project license number 33.9-42502-04-17/2571. Additionally, we complied with the ARRIVE guidelines^44^. The sound cloud experiment and the Audiobox discrimination-task experiment were conducted in Berlin, in accordance with the guidelines given by the “Landesamt für Gesundheit und Soziales Berlin (LAGESO)” and were approved by this authority. Project license numbers G173/18, G140/19.

For awake EEG experiments combined with sound conditioning, we used eleven (9 for frequency-modulated experiment, 2 for sound cloud experiment) six week-old female C57BL/6J mice (*mus musculus*) from Janvier Labs (weight range upon arrival: 15.4–17.9 g). The animals were housed in standard plastic cages with a maximum of three littermates, ad libitum food and water access. Animals were handled first by their tail and after habituation with a cupped hand. Water restriction was introduced after one week of recovery following the surgery. Moreover, the animals were kept in a 12 h/12 h light/dark cycle (5:30 am/5:30 pm) in a temperature-controlled room (~ 21 °C). Cages were enriched with nesting material, a plastic hut and a paper roll. After surgery, the paper roll was removed and the plastic hut was turned on its head to remove possible obstacles from the implanted animals. Additionally, we used eight (6 included in analysis) female C57BL/6 J mice for the discrimination task experiment in the Audiobox (TSE, Germany), where the mice lived for the duration of the experiment (see below). The amount of visits to the corner, nose pokes, consumed water and licks were analysed every day to ensure that all animals were drinking enough and in at least 50% of safe trials. Otherwise, mice were excluded from the experiment and kept under standard conditions (two out of eight animals).

### Surgery

For the implantation of the EEG drive and the EMG cable, the animal was anesthetized with either a reversible anaesthetic (2 Animals Göttingen: Medetomidine [0.5 mg/kg], Midazolam [5 mg/kg], Fentanyl [0.05 mg/kg]), or with a non-reversible one (7 Animals Göttingen: Avertin [16 mg/kg] Berlin: Ketamine [130 mg/Kg], Xylazine [10 mg/kg]). Analgesia (Göttingen: Buprenorphine [0.1 mg/kg], Berlin: Carprofen [5 mg/kg]) was injected subcutaneously as analgesic one hour before the end of the surgery. Analgesic was additionally provided subcutaneously every 24 h for the following two days. Once anesthetized, the animal was fixed in a stereo tactic frame (Kopf Instruments) and the skin above the skull was cut prior subcutaneous injection of Lidocaine ([100 mg/Kg]). Holes were drilled (drill: Osada success 40; drill tip: Fine Science Tools, size 7) into the skull with the diameter of the silver painted screws for EEG and ground. Coordinates of EEG screws with respect to Bregma (Fig. 1a): medial–lateral [− 200], anterior–posterior [− 300] (parietal screw) anterior–posterior [+ 100] (frontal screw). In an animal, we implanted parietal EEG electrodes on both hemispheres, but did not find any differences between ipsi- and contra lateral EEG respective to the speaker. To record the EMG the wire was sown through the neck muscle (*clavotrapezius*) and two knots were made to keep it in place. The screws and the EMG cable were connected to a custom-wired drive (see below). Once all connections were established, the drive was fixed to the skull with dental cement. Göttingen: anaesthesia was reversed using an antagonist: Flumazenil [0.5 mg/kg], Atipamezole [2.5 mg/kg]). The animals recovered for one week before the experiment begun.

The eight animals that were placed in the audiobox experiment underwent a minor surgery, where they were anesthetized with Ketamine-Xylazine (as above) and a small incision was made in the neck skin. A sterile transponder (PeddyMark, 12 mm × 2 mm or 8 mm × 1.4 mm ISO microchips, 0.1 gr in weight) was delivered under the skin and the skin closed with Histoacryl (B.Braun Surgical) to ensure a secure position of the transponder.

### In vivo recordings in awake animals

All awake electrical recordings were done with a chronically implanted micro drive (Göttingen: VersaDrive 4, Neurolynx, Berlin: 3D printed custom drive, Axona). EEG (stainless steel wire [203.2 µm coated diameter] with Teflon insulation, A-M Systems Inc.) and EMG (stainless steel wire [140 µm coated diameter] with PFA insulation, Science Products GmbH) wires were fed through this micro drive. The EEG was collected through a silver painted (Silberleitlack, Ferro GmbH) screw tightened in the skull over the parietal cortex (see Fig. 1a). During Recordings the micro drive was connected to an amplifier board (Göttingen: HS-36-Led, Neuralynx, USA; Berlin: RHD2132 16-Input Amplifier Board, Intan Technologies), which was connected to the acquisition system (Göttingen: Digital Lynx 4SX, Neuralynx, USA; Berlin: OpenEphys Acquisition board, OpenEphys). EEG and EMG signals were recorded (Göttingen: Cheetah Data Acquisition System software (Neuralynx, USA); Berlin: OpenEphys) at a sampling rate of 32 kHz or 30 kHz, respectively, and down sampled to 1000 Hz before analysis.

### Behavioural training on ‘audio-terrace’

The ‘audio-terrace’ is a metal construct on which the animal can move freely (see Fig. 1c, blue part). It consists of a ‘safe–terrace’, a connecting runway and a triangular corner where water access is given through two nose-poke holes. The sleeping cage of the animal was placed on the ‘safe-terrace’. In the sleeping cage, only an inverted hut and nest material was provided. The whole construct is elevated, placed on columns at a height of approximately 30 cm to restrict the animals from jumping off. In total four light barriers detected the movement of the animal over the terrace, whereby two were tracking the movement along the runway and one at each nose poke hole was tracking nose pokes. The training was automated via an analog-to-digital converter data acquisition system (NI SCB-68, National Instruments) controlled by custom written scripts (Göttingen: Matlab 2016b, Mathworks; Berlin: Python 3.6, Python Software Foundation, available at http://www.python.org). An ultrasonic speaker (Ultrasonic Dynamic Speaker Vifa, Avisoft) was placed above the nose poke corner during training on the ‘audio-terrace’ and over the sleeping cage during sleeping sessions (see Fig. 1c).

### Experimental protocol

Mice were 5 weeks of age upon arrival to the lab. After acclimation to the mouse room for at least one day, each mouse was handled 5 min daily to habituate the animal to the experimenter. After three days of this initial habituation, the implantation took place. After the surgery, the mice were allowed to recover for one week, in which they were still handled (five minutes daily). Then the experiment began. Within the first phase (habituation phase, compare Fig. 1c) the animal was trained for 20 min per day on the ‘audio-terrace’ and afterwards placed in the sleeping cage for a recording session of approximately 1.5 h. On the audio-terrace, the animal was trained to access water by poking the nose-poke-holes in the corner. During the sleep sessions, the animals habituated to being connected to the recording system and undisturbed natural sleep was recorded. Starting with the pre-exposure phase, the training protocol on the terrace was not changed, but during sleep sessions, the pre-control sound was played to habituate the animals to being exposed to sounds while sleeping. Responses to the pre-control sound were recorded for two distinct sleep states (NREM & REM sleep). After successful training (min. 500 µl water consumed during training), conditioning was introduced. During conditioning, the protocol on the audio-terrace included the timing of the start of a corner visit, and the conditioned sound was played randomly 20–40 s after the visit started. Each time the conditioned sound was played (i.e. the mouse spent enough time in the corner to trigger the sound) was counted as a trial. The conditioned sound was presented twice before an air puff and a loud sound were administered as aversive stimuli. If the mouse left the corner while the conditioned sound was still playing, the sound stopped and the trial was counted as successful, hence none of the aversive stimuli were administered. If the mouse did not escape on time, the conditioned sound was followed by punishment and the trial was counted as not successful. In Addition, the conditioned sound started to play again, continuing this loop until the mouse escaped. Mice rarely received more than two punishments per trial. Each time the mouse visited and left the corner, before the randomised timer triggered the beginning of the conditioned sound, was not considered a trial.

### Acoustic stimulation

For our experiment, we generated three to five (depending on experiment) sounds for every mouse. We used custom scripts (The Mathworks, Matlab, 2016b, or Python 3.6, Python Software Foundation, available at http://www.python.org). The sounds were created with a sampling rate of 200 kHz.

#### Pure tone—pre-control sound

The pre-control sound was a pure tone with a frequency in the centre of the other two experimental sounds and was fixed for each mouse from a range of frequencies (10–24 kHz). A pure tone was chosen in order to have a sound that was near in frequency to the conditioned and post-control sounds but not overlapping with them. Our thinking was that pure tone being absent in the natural world, would be salient and therefore a good control for the baseline effect of sound on sleep.

#### Frequency modulated sounds (chirps)—conditioned and post-control sounds

The conditioned sound was a frequency-modulated sound with an initial frequency 500 Hz above the pre-control sound and spanning at least two octaves upwards at a rate of 50 octaves/second (Fig. 1b, right). The post-control sound was also frequency modulated, but the downward mirror image of the conditioned sound. This results in relatively similar initial frequency for both the conditioned and post-control sounds and in increasing frequency differences over time. An FM sound of the given length and frequency distribution was chosen because it is known that FM sounds of these characteristics are well learned by the mice and engage auditory cortex activity. In addition this sound was, with respect to the first control, of a different nature and therefore dissociable (avoiding latent inhibition effects). The post-control sound was added to test the specificity of the effect that the conditioned sound has on ongoing sleep-oscillation. We chose another FM sound, albeit of different directionality, to make it as similar as possible in architecture to the conditioned sound, but still discriminable. The post-control sound was never presented during wakefulness because we wanted to first test the hypothesis that any sound presented during sleep once conditioning began would have an effect on sleep.

#### Sound clouds

Sound cloud is a term to describe a type of auditory stimulus characterized by multiple tones within a defined frequency range. The tones are pure tones, but due to their randomized nature, sound clouds present auditory stimuli that have increased complexity compared to continuous frequency modulated sounds and pure tones. Due to their changing frequency, sound clouds are a type of frequency-modulated sound, but differ from those used prior as they are a combination of separate pure tones rather than a continuous stimulus changing on a logarithmic scale. The sound architecture of the sound clouds used is visualized in Fig. 5a. Pre-control (3000–4662 Hz), post-control 1 (4811–7478 Hz), post-control 2 (7718–11,995 Hz), post-control 3 (12,379–19,240 Hz), and conditioned (12,379–19,240 Hz) were the five sounds used. Each sound cloud has an overall defined frequency range, or a ‘frequency window’. This window was further divided into three more narrow frequency ranges. Six pure tones were randomly selected within these three ranges, such that two tones were found within each range. These six tones were concatenated. As depicted, this overall frequency range is divided in half, and within each half, no tone is in the same frequency range as the one previously.

During sleep all sounds were played in a fixed scheme with intensities ranging from 24.6 to 73.5 dB in eight steps (Fig. 1b, left). Whether the post-control or conditioned sound was played first in a given sleep bout was randomized for each sleep phase. Additionally, also a sequence with three repetitions of zero amplitude was played at the beginning of every sound to control for possible artefacts of the speaker. Sound onset was not coupled in any way to a specific phase of sleep oscillation. Sounds were played with a free-field ultrasonic speaker (Ultrasonic Dynamic Speaker Vifa, Avisoft) through an USB audio-interface (Octa-capture, Roland) and an amplifier (Portable Ultrasonic Power Amplifier, Avisoft). Sound triggers were sent simultaneously to the I/O board of the acquisition system. Sound intensity calibration was done with a calibrated microphone (Prepolarized Free-field 1/2’’ Microphone Type 4950) and a handheld analyser (Handgehaltener Analysator Typ 2250-L, Brüel & Kjær) measuring intensities for an eight kHz pure tone. All experiments were performed in a sound-attenuated and anechoic chamber.

### Behavioural training in Audiobox

The Audiobox is an automated system to conduct unsupervised learning experiments. For a more in depth explanation and sketch see Refs.^30,43^. Briefly, the system consisted of two major parts, an experimental chamber and a home cage, which were connected via a tunnel. Food was provided ad libitum in the home-cage as well as nesting material, a hut and paper rolls, as environmental enrichment. To access water the mice had to follow a corridor to enter the drinking corner. This corner was located in a sound attenuated box and registered visits from individual animals by reading their unique transponders, which each animal was carrying underneath the neck skin. The corner had two nose poke holes, which could be closed or opened. When mice poked during safe visits the holes would open, and water access was granted. The animals were housed in a group of eight animals and could perform ad libitum on their own schedule. The amount of visits to the corner, nose pokes and licks was analysed every day to ensure that all animals were drinking in at least 50% of safe trials, as was the case for all mice. During habituation, all visits were safe, meaning that every time the mice entered the corner the safe sound was played and poking the nose poke holes would result in water access. After all animals were familiar with the drinking system, the conditioned sound was introduced in 5% of visits. In conditioning visits, the conditioned sound was played and nose poking was punished by no water access and an air puff. During the following days, the percentage of the conditioning visits was elevated to 20% or trials. After the stabilization of responses to the conditioned (nose poking during maximal 10% of conditioning trials) and safe sound (nose poking during at least 50% of safe trials), we started to introduce in total three novel sounds. In visits with novel sound, the corner was configured as for safe visits, meaning that when the mice nose poked they could access the water and no form of aversive stimulus was administered. We did not take measures to prevent mice from entering the corner together, which can lead to passive hearing the presented sound. Other studies have shown that this does not hinder the animals to generalize and discriminate between sounds^30,31^. We calculated the response of the animals to each of the experimental sounds by checking the percentage of visits in which there was a nose poke for each of the experimental sounds.

### Data analysis and statistics

The analysis and statistics of the electrophysiology and behavioural data was done in a Matlab/Python environment. For the sleep analysis without sound presentation, the power was extracted from the parietal EEG-trace with the fast-Fourier transformation for frequencies of 0–4 Hz for NREM sleep and 6–10 Hz for REM sleep in a time window of 1 s for the 8 s before, during sound presentation and the 8 s afterwards. The resulting power data was normalized by the mean power across the 8 s prior to sound presentation for each recording. The peak-to-peak variability during NREM and REM sleep was calculated by identifying all peaks (positive and negative) in the raw EEG trace and then evaluate the standard deviation for absolute peak amplitude values.

For the sleep analysis with presentation of the experimental sounds, the power was calculated and normalized in the same way as described above. Afterwards all normalized recording were first averaged over all recordings of each animal and afterwards over all animals.

The statistical analysis was conducted using linear mixed models, to account for the repeated measures of the experimental design. For the analysis of the audiobox data we used an ANOVA and Tukey–Kramer post-hoc multiple comparison test.

## Supplementary Information

Supplementary Information.

## Data Availability

The datasets generated during and/or analysed during the current study are available from the corresponding author on reasonable request.
