# Supplementary material for: Sound disrupts sleep-associated brain oscillations in rodents in a meaning-dependent manner
Source: Sci Rep. 2022 Apr 11;12:6051. doi: 10.1038/s41598-022-09457-6 (PMC9001723; doi:10.1038/s41598-022-09457-6)
Supplement: Supplementary file 1 — Supplementary Information. [file 41598_2022_9457_MOESM1_ESM.pdf]

Supplementary Figure 1. Power boxplots for extended frequency ranges during NREM and REM sleep

(a+b) Comparison of the normalized and averaged power over time during **(a)** NREM and **(b)** REM sleep. Power is calculated for five frequency ranges each second, over a 24-second period (eight seconds, before, during and after sound presentation). Outliers over a power level of 4 are exculded.

Suppl. 1

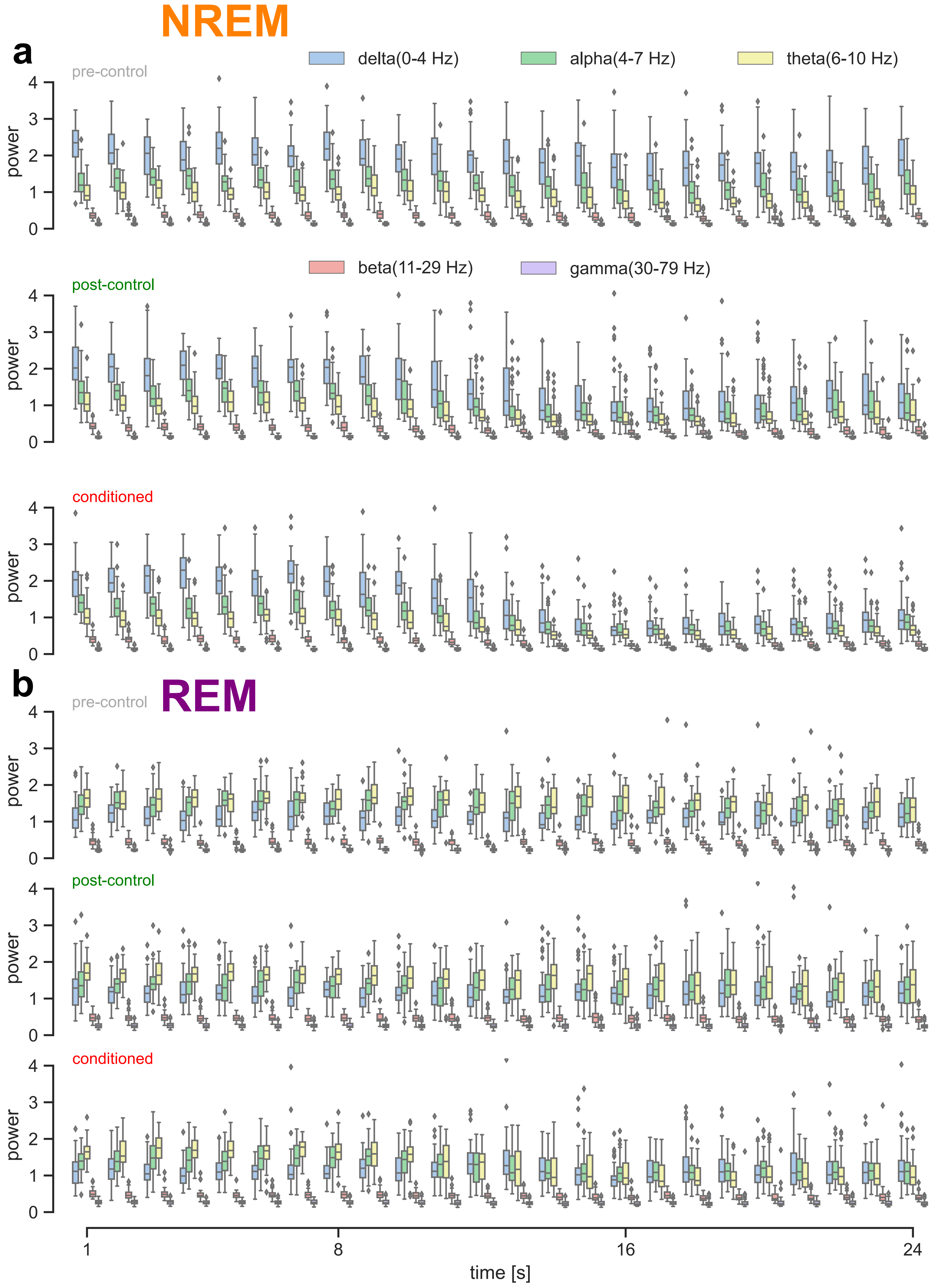
NREM
a
delta(0-4 Hz)
alpha(4-7 Hz)
theta(6-10 Hz)
beta(11-29 Hz)
gamma(30-79 Hz)
pre-control
post-control
conditioned
power
b
REM
pre-control
post-control
conditioned
power
1
8
16
24
time [s]
